# Spatio-Temporal Distribution and Fixed-Precision Sampling Plan of *Scirtothrips dorsalis* (Thysanoptera: Thripidae) in Florida Blueberry

**DOI:** 10.3390/insects12030256

**Published:** 2021-03-18

**Authors:** Babu R. Panthi, Justin M. Renkema, Sriyanka Lahiri, Oscar E. Liburd

**Affiliations:** 1 Gulf Coast Research and Education Center, University of Florida, Wimauma, FL 33598, USA; justin.Renkema@canada.ca (J.M.R.); lahiris@ufl.edu (S.L.); 2 Crop and Soil Science Department, Oregon State University, Corvallis, OR 97331, USA; 3 London Research and Development Centre-Vineland Campus, Agriculture and Agri-Food Canada, Vineland, ON L0R 2E0, Canada; 4 Department of Entomology and Nematology, University of Florida, Gainesville, FL 32611, USA; oeliburd@ufl.edu

**Keywords:** chilli thrips, invasive pest, spatial analysis, aggregation indices, monitoring

## Abstract

**Simple Summary:**

*Scirtothrips dorsalis*, chilli thrips, is an invasive insect species in Florida and an important foliar pest of blueberry. Sound knowledge of insect distribution within the field is needed to formulate accurate sampling methods. Fourteen blueberry fields were systematically sampled for chilli thrips during the summers of 2017 and 2018. Field counts were modeled in various spatial models and determined chilli thrips had temporally stable aggregated distribution. A fixed-precision sampling plan was developed for summer sampling, requiring seven and three sampling units (sampling unit = 10 young blueberry shoots) to estimate a nominal mean density of 20 chilli thrips with a precision of 25% and 40%, respectively. The sampling plan can be used to improve the timing of control measures and assess the effectiveness of these control measures.

**Abstract:**

*Scirtothrips dorsalis* Hood is an invasive and foliar pest of Florida blueberry that reduces plant growth by feeding on new leaf growth. A sampling plan is needed to make informed control decisions for *S. dorsalis* in blueberry. Fourteen blueberry fields in central Florida were surveyed in 2017 and 2018 after summer pruning to determine the spatial and temporal distribution of *S. dorsalis* and to develop a fixed-precision sampling plan. A sampling unit of ten blueberry shoots (with four to five leaves each) was collected from one blueberry bush at each point along a 40 × 40 m grid. Field counts of *S. dorsalis* varied largely ranging from zero to 1122 adults and larvae per sampling unit. *Scirtothrips dorsalis* had aggregated distribution that was consistent within fields and temporally stable between summers, according to Taylor’s power law (TPL) (aggregation parameter, *b* = 1.57), probability distributions (56 out of 70 sampling occasions fit the negative binomial distribution), Lloyd’s index (*b* > 1 in 94% occasions), and Spatial Analysis by Distance IndicEs (31% had significant clusters). The newly developed fixed-precision sampling plan required 167, 42, seven, or three sampling units to estimate a nominal mean density of 20 *S. dorsalis* per sampling unit with a precision of 5%, 10%, 25%, or 40%, respectively. New knowledge on *S. dorsalis* distribution will aid in evaluating the timing and effectiveness of control measures.

## 1. Introduction

*Scirtothrips dorsalis* Hood (Thysanoptera: Thripidae) is invasive to the United States [1], as it was accidentally introduced from South Asia and became established in south Florida in 2005 in Knock Out^®^ rose plants (*Rosa* X ‘Radrazz’: Rosaceae) [2]. Since then, *S. dorsalis* has become a pest of vegetable, ornamental, and fruits crops, and minor pest of landscape plants in Florida and Texas [1,3,4,5,6,7]. It has also been reported in other southern US states including Louisiana, Georgia, Alabama, Mississippi, and South Carolina, with a potential to expand up to western and northeastern US states and southern Canada [1,8,9]. In Florida, *S. dorsalis* is a significant pest of pepper (*Capsicum* spp., Solanaceae) [10], rose [11], strawberry (*Fragaria × ananassa* Duchesne, Rosaceae) [12], blueberry (*Vaccinium* sp., Ericaceae) [13], and landscape plants [5]. *Scirtothrips dorsalis* is minute (1–2 mm long) with yellow-colored body, dark-fringed wings, and dark stripes on dorso-abdominal segments [14]. Adult females live for 18–25 days at 25 °C and oviposit 40–50 kidney-shaped eggs underneath leaf tissues [15]. Larvae with transparent white bodies eclose in 67 days and complete two actively feeding instars in 4–6 days at 25 °C [16]. Two inactive pupal stages complete development either on leaf, leaf litter, or soil in 2—3 days at 25 °C [4]. Both adults and larvae cause injury to new leaf growth by removing cellular contents from leaf tissues using piercing and sucking type mouthparts.

*Scirtothrips dorsalis* occurs year-round in perennial blueberry in Florida and is abundant on new leaf growth after summer pruning [4]. Feeding injury on blueberry plants first appears as bronzing along leaf veins and blades, and heavy *S. dorsalis* infestation causes upward leaf curl and distortion [4]. Hot and humid summers favor rapid population growth of *S. dorsalis,* leading to a reduction of blueberry growth. Insecticides are routinely applied to control *S. dorsalis* in conventional fields [13], although other eco-friendly control measures are available such as mycoinsecticides and biological control agents [1]. Insecticides are usually applied every two weeks (B. Panthi pers. comm.), as the highly effective insecticide, spinetoram, has a residual activity for about two weeks [13]. When insecticides with the same mode of action are applied repeatedly, there is increased selection pressure for thrips populations to develop resistance [17]. *Scirtothrips dorsalis* has developed resistance to monocrotophos, acephate, dimethoate, phosalone, carbaryl, and triazophos in India [17]. In another study, *S. dorsalis* showed no or very low level of resistance against organophosphates (acephate, chlorpyrifos, quinalphos, and dimethoate), neonicotinoids (thiamethoxam, imidacloprid), and abamectin as measured by resistance ratio (RR) but showed 21–28 fold increase in RR to Spinosad 45SC probably due to multiple applications [18]. Moreover, frequent insecticide applications negatively affect non-target organisms that help to regulate thrips populations in blueberry plantings [19,20]. Thus, for sustainable management of *S. dorsalis*, insecticides should be judiciously applied.

Sampling plans are important tools for integrated pest management as they improve the decision-making process by accurately estimating the pest population in the field [21] so that unnecessary insecticide applications are avoided. Use of these plans typically requires fewer samples and thus less time to estimate pest populations compared to whole field scouting [21]. In addition, such tools can be used to evaluate the effectiveness of control measures [10,11,22]. A prerequisite of a sampling plan is the determination of the field spatial distribution of a pest by systematically sampling the pest [23]. Field pest counts can then be modeled in spatial and non-spatial models to determine the distribution parameters and aggregation indices specific to a pest in a given crop [23]. Finally, the distribution parameters can be modeled with mathematical equations to calculate the number of sampling units required to estimate a pest’s mean field density [23]. Sampling plans of *S. dorsalis* have been developed for pepper and rose, by systematically collecting young leaf samples, counting the number of *S. dorsalis* per sampling unit, and calculating and modeling the distribution parameters [10,11,22]. However, since the distribution parameters and aggregation indices of an insect species vary between crops, the number of sampling units required to estimate the mean density changes by crop [23]. The information on the distribution patterns of *S. dorsalis* is lacking for Florida blueberry. Therefore, there is a considerable opportunity to improve the monitoring and decision of *S. dorsalis* in blueberry fields. The objectives of this study were to determine the spatial and temporal distribution of *S. dorsalis* in blueberry fields and to develop a fixed-precision sampling plan.

## 2. Materials and Methods

### 2.1. Field Surveys

Fourteen southern highbush blueberry fields were surveyed from Jun to Sep in 2017 and 2018 in central Florida to assess the spatial and temporal distribution of *S. dorsalis* (Table 1). All but one field had one or two cultivars (Table 1). Growers pruned lateral shoots about two weeks before we first sampled, and most fields received two or three applications of insecticides during the summers (Table 2). A few growers were unwilling to share pruning and insecticide information and so information of only eight fields are presented in Table 2. Ten young blueberry shoots (four or five leaves each) were collected from one blueberry bush as one sampling unit per grid point along 40 × 40 m grids in every field. The first grid point in each field was 1.5 m from the edges near a field corner; its GPS coordinates were recorded. Due to variable field sizes (1.6–5.9 ha), the number of sampling units varied from 18 to 45 per field. Each field was sampled three times each year, with intervals of at least 25 days. Sampling occurred during the morning hours (900–1100 h) when *S. dorsalis* activity was low [24]. Leaf samples were placed in labeled plastic sealed bags (15 × 12 cm, Ziploc^®^, S.C. Johnson and Son, Inc., Racine, WI, USA) and brought back to the laboratory. Two hundred and fifty mL of 70% ethanol was poured into the bag. Leaf samples were rinsed by manually shaking the bag for two minutes and the solution was poured into a 500 mL volume plastic container. The leaves were further rinsed, the solution with *S. dorsalis* was poured through a 160-micron mesh cloth, and the filtrate with *S. dorsalis* was transferred onto a 10 × 10 cm gridded petri-dish (FB0875711A, Fisherbrand^TM^, Thermo Fisher Scientific, Waltham, MA, USA). The Petri dish with the filtrate was then observed under a stereomicroscope (LEICA LED3000 SLI, Leica Microsystems Inc, Wetzlar, Germany) at 25× magnification, and both stages of *S. dorsalis* were identified [25].

### 2.2. Statistical Analysis

The location of grid points of each field was categorized as either ‘inside of the field’ or ‘field edge’ and grid points designated as ‘field edge’ were subcategorized as adjacent to ‘vegetation space’ or ‘open space’. Then, the probability of *S. dorsalis* occurring in each category was assessed with contingency tables, using the likelihood ratio tests. Data were log (*x* + 1) transformed to normalize the error variance. Tukey’s honest significance test was used as a *post-hoc* mean separation at *P* = 0.05, and the back-transformed means and 95% confidence intervals are presented. Analyses were made using JMP^®^ Pro 15.0.0 (SAS Institute Inc., Cary, NC, USA).

### 2.3. Spatial Analysis

Field means and variances of *S. dorsalis* counts were modeled in Taylor’s power law (TPL) using the *power law* function. Taylor’s power law relates variance ($\sigma^{2}$) of counts per unit area to the corresponding mean (μ) by a power law such that,

(1) $$\sigma^{2}=a\mu^{b}$$

where *a* is a sampling factor and *b* is an aggregation parameter. The relationship is linearized as,

log(*V*) = log(*a*) + *b* * log(*M*) (2)

where the sample mean (*M)* approximates the population mean (μ), the sample variance (*V*) approximates the population variance ($\sigma^{2}$), log(*a*) is the intercept, and *b* is the slope or the aggregation parameter. The distribution is considered random if *b* = 1, regular if *b* < 1, and aggregated if *b* > 1. Field counts were also fit to the Poisson distribution and Negative binomial distribution using the *fit_two_distr* function, and data were considered to belong to a frequency distribution when the observed and expected frequencies were not different (Chi-square test at *p* > 0.05). The function *sadie* was used to calculate the index of SADIE (Spatial Analysis of Distance Indices) index, $I_{a},$ which indicates significant clustering if the index is greater than 1.5 [26,27]. Lloyd’s patchiness index, *P,* was determined using the function *agg index*, and the degree of patchiness increases as the index becomes greater than the unity. The R-package *epiphy* was used for all the spatial analyses using R, version 3.5.1. Correlations between aggregation indices ($I_{a}$, *k*, and *P*), sampling dates within and between years, and field means were determined through multivariate analysis in JMP^®^ Pro 15.0.0 (SAS Institute Inc. 2018).

### 2.4. Sampling Plan

To calculate a fixed-precision sampling plan, TPL parameters (*a* and *b*) were modeled in Equation (1) for each *S. dorsalis* mean density, m. Then, the number of sampling units (*n*) required to estimate a range of mean densities with 5%, 10%, 25%, and 40% coefficient of variation (*CV*) was calculated by inserting *v* and *m* in Equation (3) described by Binns et al. [23]:

(3) $$n=v{\left(\frac{1}{m*CV}\right)}^{2}$$

## 3. Results

### 3.1. Field Surveys

**Figure 1:**
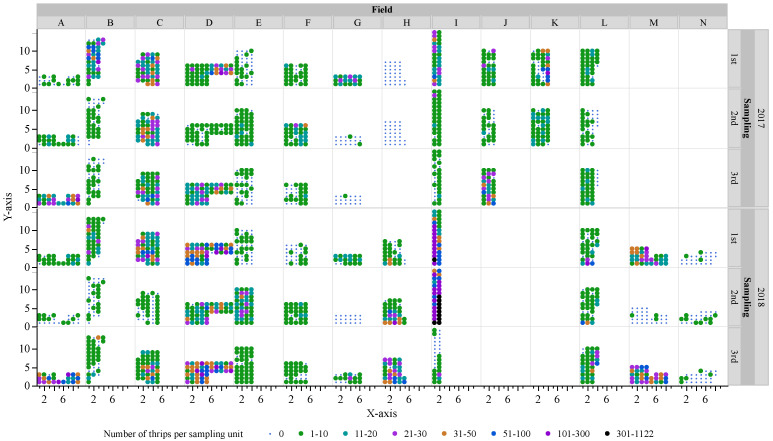
Field counts of *Scirtothrips dorsalis* per sampling unit (ten blueberry shoots) collected from fourteen blueberry fields in central Florida during summers of 2017 and 2018. Each dot represents a blueberry bush (one sampling unit) in 40 × 40 m grids.

From 70 sampling occasions, a total of 35,631 *S. dorsalis* were sampled of which 23,818 were larvae and 11,813 were adults. The number of *S. dorsalis* ranged from 0–857 larvae and 0–442 adults per sampling unit with a mean of 10.4 larvae and 5.2 adults per field per sampling unit. Among 2282 sampling units, 46% had one to ten *S. dorsalis*, 25% had no *S. dorsalis*, 24% had 10–50 *S. dorsalis*, and 5% had 50–1122 *S. dorsalis* per sampling unit (Figure 1). Among 70 sampling occasions, 19% had field means of zero to one *S. dorsalis*, 66% had field means of one to 20 *S. dorsalis*, 13% had field means of 20–60 *S. dorsalis*, and 1% had field means of 60–333 *S. dorsalis*. The field ‘I’ had the highest mean density of 333 *S. dorsalis* per sampling unit with counts up to 1122 *S. dorsalis* per sampling unit during the second and third sampling date in 2018.

The likelihood ratio test showed *S. dorsalis* occurred equally on the field edge (53%) or inside of the field (47%) (Table 3). However, when tested (likelihood ratio test) among those occurring along field edges, there were more *S. dorsalis* near open spaces (64%) than near surrounding vegetation (36%) (Table 3).

### 3.2. Spatial Analysis

**Figure 2:**
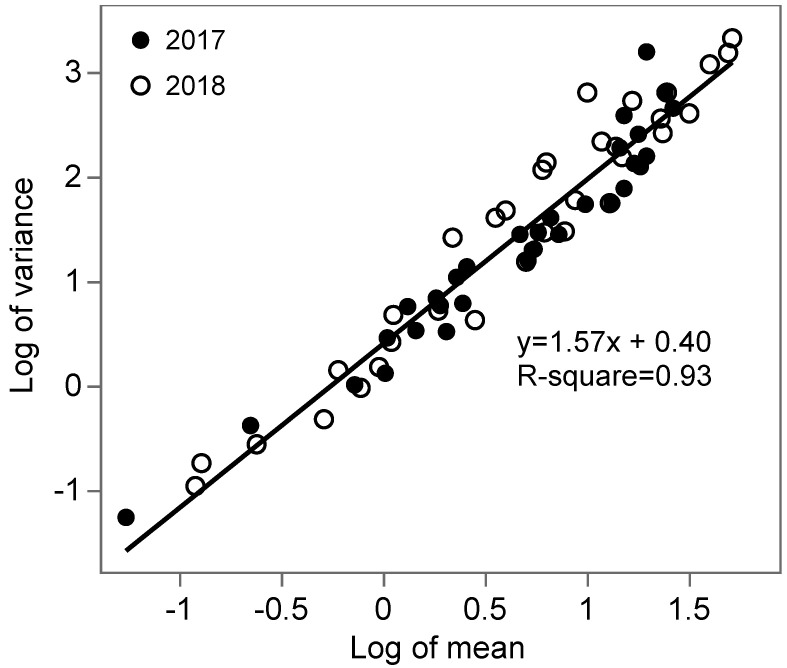
Linear relationship between field variances and means (*n* = 70) of *S. dorsalis* counts from leaf samples collected from fourteen blueberry fields in central Florida during summers of 2017 and 2018.

**Figure 3:**
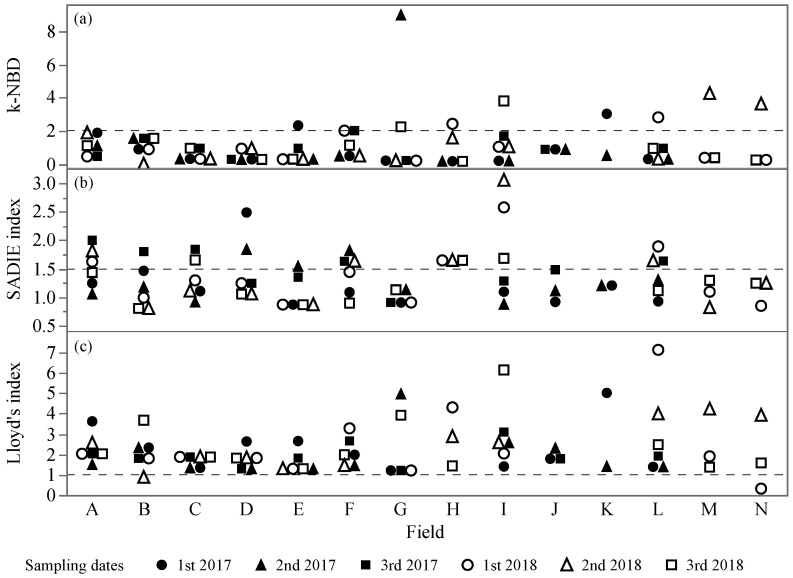
Aggregation indices from adult and larval *S. dorsalis* count on leaf samples in fourteen blueberry fields in central Florida during the summers of 2017 and 2018. Dashed lines are the break-even points of aggregation indices; the distribution of the counts is aggregated if (**a**) k-NBD (Negative Binomial Distribution) < 2, (**b**) SADIE (Spatial Analysis of Distance Indices) index > 1.5, and (**c**) Lloyd’s index > 1.

Variances and means were significantly related as shown by Taylor’s power law (*r*^2^ = 0.93, *p* < 0.0001, *a* = 1.50, *b* = 1.57), indicating an aggregated distribution of *S. dorsalis* in blueberry fields (Figure 2). Fifty-six out of 70 sampling occasions fit the NBD, three fit the Poisson distribution, four fit both, and seven fit neither distribution (Chi-square test: *p* > 0.05). For counts that fit the NBD, 46 had *k* values below two, indicating highly aggregated populations (Figure 3a). Twenty-two out of 70 sampling occasions had SADIE indices greater than 1.5 (Figure 3b). A significant positive correlation between the SADIE index and field means (*r*^2^ = 0.56, *p* < 0.0001) showed that the clustering of *S. dorsalis* increased with the *S. dorsalis* field mean density. Sixty-eight out of 70 sampling occasions had Lloyd’s index greater than the unity, indicating significant patches of *S. dorsalis* populations (Figure 3c). Non-significant correlations between aggregation indices (*k*, $I_{a}$, and *P*) and sampling dates within year (*k*: *r*^2^ = −0.002, *p* = 0.98; $I_{a}$: *r*^2^ = 0.06, *p* = 0.61; and *P*: *r*^2^ = −0.05, *p* = 0.69) and between years (*k*: *r*^2^ = 0.03, *p* = 0.83; $I_{a}$: *r*^2^ = 0.01, *p* = 0.93; and *P*: *r*^2^ = 0.16, *p* = 0.19) showed that the *S. dorsalis* distribution was temporally stable within fields and between seasons, respectively. The field mean density of *S. dorsalis* also did not change with sampling dates within the same year (*r*^2^ = −0.03, *p* = 0.8) and between the two years (*r*^2^ = 0.19, *p* = 0.11).

### 3.3. Fixed-Precision Sampling Plan

**Figure 4:**
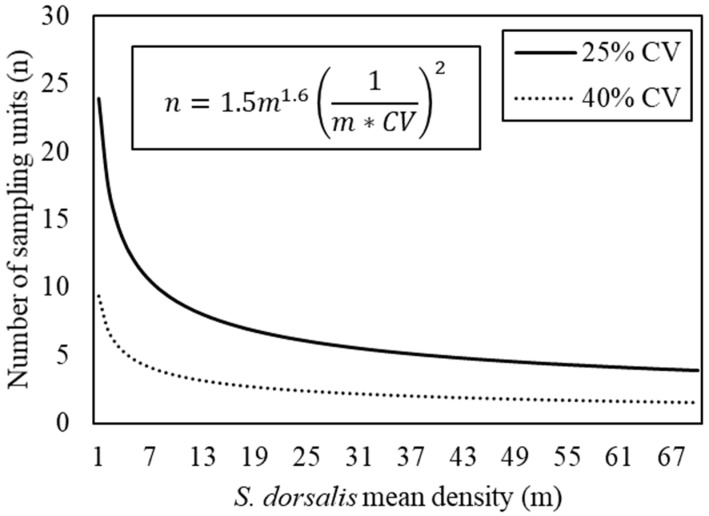
Number of sampling units (n) required to estimate the *S. dorsalis* mean density (m) in Florida blueberry fields with variable coefficients of variation (CV) calculated by modeling Taylor’s power law parameters into the mathematical formula: $n={a*m}^{b}{\left(1/m*CV\right)}^{2}$.

The number of sampling units required to estimate the *S. dorsalis* population decreased as CV and/or mean density increased (Figure 4). According to the fixed-precision sampling plan, it would require 167, 42, seven, or three sampling units to estimate a nominal mean density of 20 *S. dorsalis* per sampling unit with a CV of 5%, 10%, 25%, or 40%, respectively. Although sampling units can be calculated for any precision level by solving the equation provided in Figure 4, the figure only presents sampling plans for 25% and 40% precisions for a clear figure.

## 4. Discussion

This is the first study to characterize the spatial and temporal distribution of *S. dorsalis* in blueberry fields in Florida. Spatial and non-spatial models fit to the data showed a consistent aggregated distribution of *S. dorsalis* in blueberry fields. The distribution of *S. dorsalis* in particular fields was stable between seasons, allowing sampling plans to be used throughout the season in blueberry fields. The fixed-precision sampling plan developed as a result of characterizing the field distribution of *S. dorsalis* requires three and seven sampling units (one sampling unit = 10 blueberry shoots) to estimate a nominal mean density of 20 *S. dorsalis* per sampling unit (two *S. dorsalis* per blueberry shoot) with a precision of 25% and 40%, respectively. In other crops affected by S. dorsalis, seven or nine leaf samples were required to estimate a mean density of one *S. dorsalis* in rose with a precision of 25% and two *S. dorsalis* in pepper with a precision of 10%, respectively [10,11]. Sampling for *S. dorsalis* along blueberry field edges next to open space will maximize the accuracy of the sampling plan since more *S. dorsalis* were found at edge locations next to open than vegetated space.

The aggregated distribution of *S. dorsalis* in blueberry fields is likely due to minimal movement of *S. dorsalis* among blueberry bushes [25]. *Scirtothrips dorsalis* showed similar behavior in strawberry where adults moved slowly between strawberry plants and were aggregated in strawberry fields [25]. The aggregated behavior of *S. dorsalis* was also observed in pepper fields [10] and potted rose plants [24]. The slow movement and aggregation of *S. dorsalis* are likely to be due to aggregation pheromones as in other thrips, *Frankliniella occidentalis* (Pergande), and *Thrips palmi* Karny (Thripidae) [28]. Therefore, there is an opportunity to test for the presence and potency of the sex pheromone of *S. dorsalis* to advance the knowledge of *S. dorsalis* biology and develop potential pest management techniques such as mass trapping.

In this study, as mean density increased, the area of *S. dorsalis* clusters (SADIE index) increased, which is likely due to outward movement of *S. dorsalis* from the initial point of infestation [29]. However, a significant increase in the cluster area was found only when the mean density was higher than 50 *S. dorsalis* per ten blueberry shoots. Assuming that plants with defoliated blueberry leaves become poor hosts for adult and larval thrips, enhanced movement of adults in response to a deterioration of blueberry shoots is expected to increase their lifetime reproductive success. However, since the quantity and nutritional quality of healthy and defoliated leaves as food resources was not assessed in this study, this hypothesis remains to be tested experimentally. Future studies should focus on evaluating whether and to what extent aggregations of *S. dorsalis* on host plants affect the survivorship and fecundity of *S. dorsalis.*

Blueberries in Florida are pruned during summer after harvest to promote new growth. Summer pruning may disrupt *S. dorsalis* populations in blueberry and reduce population build-up. We did not test the effect of pruning timing; however, future studies should test whether delayed pruning could reduce the impact of *S. dorsalis* in blueberry. In addition, the shoots infested with *S. dorsalis* moved to healthy shoots of grapes in Japan [30]. Therefore, the pruned shoots should be properly disposed of to reduce further infestations as *S. dorsalis* might disperse out from the pruned and defoliated leaves.

For estimating *S. dorsalis* populations in blueberry fields, we recommend collecting ten young blueberry shoots per bush (=one sampling unit) and counting all larvae and adults. Seven sampling units are required to estimate a nominal mean density of 20 *S. dorsalis* per sampling unit with a precision of 25%. A precision of 25% is acceptable in estimating mean densities of insects in the field, whereas a precision of 10% is acceptable for research purposes [23]. However, a lower precision of 40% is sufficient when estimating mean density to evaluate the insecticide efficacy. At a precision level of 40%, just three sampling units are needed to estimate the mean density.

Numbers of sampling units required to estimate *S. dorsalis* mean density in blueberry varied from other crops such as strawberry, rose, and pepper, where *S. dorsalis* infested heavily on new leaf growths. The difference in the number of sampling units is likely related to how *S. dorsalis* is distributed among host plants. Thrips have aggregated distributions within fields, however, aggregation patterns vary among plant species because of how they distribute among plants. Such aggregations are characterized using aggregation indices such as slope parameter of TPL model, which increases with aggregation [31]. The TPL parameter ‘b’ of *S. dorsalis* in blueberry was 1.57 and was 1.72 in strawberry, indicating a more aggregated population of *S. dorsalis* in strawberry than blueberry fields. A recent study showed that *S. dorsalis* move slowly between strawberry plants, causing the population to aggregate in initially infested plants. Similarly, the aggregation index in rose was 1.14 (lesser aggregation than in blueberry and strawberry), and it varied from 1.10 to 1.63 in pepper fields with respect to different plot sizes.

A fixed-precision sampling plan will allow the estimation of *S. dorsalis* populations with a manageable number of sampling units. Estimating a mean density of 20 *S. dorsalis* per sampling unit would require 5 min to collect ten blueberry shoots and count the number of *S. dorsalis* in blueberry fields, for a total of 35 min to collect seven sampling units. Future research is needed to determine an economic threshold of *S. dorsalis* to make accurate control decisions to manage this pest in blueberry fields. An economic threshold requires a direct relationship between pest density and plant growth or yield. The *S. dorsalis* population levels in blueberry fields in the summer may not necessarily have a direct relationship to fruit harvests the following spring (April–May). However, *S. dorsalis* infestations over time can weaken bushes, reduce the growth, and ultimately affect the yield. Therefore, two thresholds and sequential sampling plans may be needed to time insecticide application, one to avoid injury during the summer when plants are young and a second pre-harvest threshold for older, bearing bushes to avoid yield losses.

## 5. Conclusions

*Scirtothrips dorsalis* heavily infests new blueberry shoots after summer pruning and reduces plant growth by leaf defoliation. New knowledge on the distribution of *S. dorsalis* in fields resulting in improved sampling methods will enable better management decisions and more profitable blueberry production in Florida. However, management actions with insecticides should be timed in a way as not to disrupt the ecosystem services and only if the threshold is high enough to cause yield reduction.

## Figures and Tables

**Table 1:** Location, characteristics, and sampling dates of blueberry fields (14) in central Florida where young blueberry shoots were sampled randomly for *S. dorsalis* during summers of 2017 and 2018.

Field	GPS Coordinates		Florida County	No. of Samples	Field Area (ha)	Cultivars	2017 Sample Dates			2018 Sample Dates		
	Latitude	Longitude					1st	2nd	3rd	1st	2nd	3rd
A	27.95216	−82.15062	Hillsborough	23	2.4	mdl, jwl	20 June	24 July	30 August	20 June	24 July	27 August
B	27.95217	−82.13344	Hillsborough	37	3.5	jwl, emr, ksr, ckd, sph	20 June	1 August	31 August	20 June	24 July	27 August
C	27.95160	−82.12887	Hillsborough	41	4.8	fth	21 June	27 July	31 August	27 June	26 July	27 August
D	27.94213	−82.14359	Hillsborough	45	5.9	ckd	21 June	24 July	30 August	27 June	26 July	27 August
E	27.73754	−82.22836	Hillsborough	40	4.7	ksr	22 June	26 July	29 August	22 June	27 July	14 September
F	27.74114	−82.23246	Hillsborough	30	3.5	vnt, fth	22 June	26 July	29 August	22 June	27 July	14 September
G	27.82484	−82.18275	Hillsborough	18	1.6	jwl, emr	23 June	27 July	29 August	5 July	24 July	7 September
H	28.75730	−82.27129	Citrus	30	3.1	emr	19 June	26 July	x	3 July	3 August	5 September
I	28.42770	−81.78181	Lake	30	2.8	emr	19 June	28 July	30 August	3 July	3 August	5 September
J	28.44823	−81.68027	Lake	30	2.9	emr	20 June	31 July	30 August	x	x	x
K	28.38861	−82.34166	Pasco	40	4.7	emr	27 June	2 August	x	x	x	x
L	28.44956	−82.34541	Hernando	35	4.2	emr	30 June	26 July	30 August	3 July	3 August	5 September
M	27.69529	−82.15803	Hillsborough	31	2.9	jwl, emr	x	x	x	5 July	24 July	14 September
N	28.74367	−82.27950	Citrus	25	3.0	swt	x	x	x	3 July	3 August	5 September

Cultivar abbreviations: mdl = Meadowlark, jwl = Jewel, emr = Emerald, ksr = Kestrel, ckd = Chickadee, sph = Spring High, fth = Farthing, vnt = Ventura, swt = Sweet Crisp. (x) information not available.

**Table 2:** Pruning and insecticide application dates for seven blueberry fields (code: A–G) in Hillsborough County, FL and one field (code: L) in Hernando County, FL, where young blueberry shoots were sampled to assess the distribution of *S. dorsalis* during summers of 2017 and 2018.

Field Code	Pruning Dates		Insecticide Application Dates											
	2017	2018	2017						2018					
			1st		2nd		3rd		1st		2nd		3rd	
A	12 June	6 June	*		27 July	fen †	21 August	fen	13 June	cya	27 July	cya	18 August	kao
B	1 June	30 May	16 July	ace	26 July	fen	19 August	spi + nov	*		*		18 August	spi + nov
C	30 May	1 June	1 July	cya	15 August	mal	30 August	mal	1 July	cya	-		-	
D	15 May	18 May	7 June	spi	17 July	spi	*		14 June	ace	14 July	ace	6 August	bif
E	1 June	1 June	1 July	cya	15 August	mal	30 August	mal	1 July	cya	*		18 August	spi
F	1 June	1 June	1 July	cya	15 August	mal	30 August	mal	1 July	cya	*		18 August	spi
G	30 May	30 May	4 July	spi + nov	8 July	bif	20 August	spi + ace	2 July	spi + ace	*		-	
L	-	-	-		-		-		*		*		15 August	spi

(*) no spray. † Information on product’s active ingredient (a.i.) and IRAC (Insecticide Resistance Action Committee) mode of action code: fen = fenpropathrin; 3A, cya = cyantraniliprole; 28, kao = kaolin; OMRI listed, ace = acetamiprid; 4A, spi + nov = spinetoram + novaluron; 5 + 15, spi = spinetotam; 5, bif = bifenthrin; 3A, mal = malathion; 1B, spi + ace = spinetoram + acetamiprid; 5 + 4A. (-) information not available

**Table 3:** Occurrences of *S. dorsalis* on blueberry plantings, inside of field or near field edges and with open space or surrounding vegetation, in central Florida during the summers (June–September) of 2017 and 2018.

Occurrence	Location		Field Edge	
	Inside of Field	Field Edge	Open Space	Surrounding Vegetation
No (=0 *S. dorsalis)*	248	315	170	145
Yes (>0 *S. dorsalis)*	828	891	598	293
Likelihood ratio test	Chi-square = 2.9, *p* = 0.089		Chi-square = 17.07, *p* < 0.001	

## Data Availability

Not applicable.
